# Differential Impact of Acute and Chronic Stress on CA1 Spatial Coding and Gamma Oscillations

**DOI:** 10.3389/fnbeh.2021.710725

**Published:** 2021-07-20

**Authors:** Anupratap Tomar, Denis Polygalov, Thomas J. McHugh

**Affiliations:** Laboratory for Circuit and Behavioral Physiology, RIKEN Center for Brain Science, Saitama, Japan

**Keywords:** hippocampus, acute stress, chronic stress, place cells, theta, slow gamma, fast gamma, phase-locking

## Abstract

Chronic and acute stress differentially affect behavior as well as the structural integrity of the hippocampus, a key brain region involved in cognition and memory. However, it remains unclear if and how the facilitatory effects of acute stress on hippocampal information coding are disrupted as the stress becomes chronic. To examine this, we compared the impact of acute and chronic stress on neural activity in the CA1 subregion of male mice subjected to a chronic immobilization stress (CIS) paradigm. We observed that following first exposure to stress (acute stress), the spatial information encoded in the hippocampus sharpened, and the neurons became increasingly tuned to the underlying theta oscillations in the local field potential (LFP). However, following repeated exposure to the same stress (chronic stress), spatial tuning was poorer and the power of both the slow-gamma (30–50 Hz) and fast-gamma (55–90 Hz) oscillations, which correlate with excitatory inputs into the region, decreased. These results support the idea that acute and chronic stress differentially affect neural computations carried out by hippocampal circuits and suggest that acute stress may improve cognitive processing.

## Introduction

It is generally accepted that while mild or acute stress can be beneficial for cognition and learning, repeated exposure to stressors (chronic stress) disrupts these processes (Luksys and Sandi, 2011). This dichotomy in the impact of acute and chronic stress has also been observed in the hippocampus, a brain region crucial for the acquisition and consolidation of declarative memory. At the cellular level, chronic, but not acute stress, causes dendritic shrinkage and debranching (Watanabe et al., 1992; Sousa et al., 2000) and decreases the number of synaptic contacts (spines) on principal hippocampal pyramidal neurons (Magariños et al., 1997; Sandi et al., 2003). Further, earlier studies employing both *ex vivo* electrophysiology and *in vivo* tetrode recordings report that chronic stress also alters the functionality of hippocampal pyramidal cells. For example, chronic stress disrupts synaptic plasticity in hippocampal slices (Alfarez et al., 2003). Similarly, the spatial map or internal representation of the surroundings (O’Keefe and Nadel, 1978), evident in the location-specific increase in average firing rate of hippocampal pyramidal “place” cells (O’Keefe and Dostrovsky, 1971; O’Keefe, 1976) is altered in chronically stressed rodents (Kim et al., 2007; Passecker et al., 2011; Tomar et al., 2015). However, the interpretation of acute stress effects on hippocampal synaptic plasticity is more complex (Joëls and Krugers, 2007; MacDougall and Howland, 2013) and consequently, the impact of acute stress on the neural computations carried out by hippocampal circuits in the intact brain remains unclear.

In addition to the rate code (i.e., location-specific spiking), place cells also use temporal coding to signal spatial aspects of the animal’s location or behavior (O’Keefe and Recce, 1993). Temporal coding involves place cells spiking at a specific phase of ongoing oscillations in the local field potential (LFP), such as theta (6–12 Hz) and gamma (30–90 Hz), during exploratory behavior. These oscillations, as well as the more transient coupling of the theta-gamma oscillations themselves, are thought to provide temporal precision to the activity of hippocampal cell assemblies and to facilitate phenomena including synaptic plasticity and retrospective and prospective coding (Harris et al., 2003; Lisman, 2005; Buzsáki, 2010; Fries, 2015). Interestingly, temporal coding, as well as theta and gamma coupling, have been shown to be altered in neurodegenerative disorders (Goutagny et al., 2013; Booth et al., 2016; Mably et al., 2017), for which stress is a risk factor (Bisht et al., 2018). Thus, it is likely that both acute and chronic stress may impact these oscillatory patterns in unique ways.

To address these gaps in our knowledge we employed tetrode recordings in the dorsal CA1 of male mice. Recordings were made while mice explored a linear track before and after experiencing chronic immobilization stress (CIS; Suvrathan et al., 2010), a protocol that has been previously shown to reduce hippocampal volume, spatial memory (Rahman et al., 2016), and context discrimination (Tomar et al., 2015). Specifically, we examined alterations in both rate and temporal coding of CA1 pyramidal cells, as well as changes in the hippocampal oscillatory activity, following acute and chronic stress.

## Materials and Methods

### Animals

All experiments were performed using male C57BL/6J mice. A total of five mice, aged between 3 and 6 months, were used for this study. The data related to the physiology during the stress exposure from these mice was previously reported (Tomar et al., 2021). Mice were maintained on a 12-h light-dark cycle with *ad libitum* access to food and water. All procedures were approved by the RIKEN Institutional Animal Care and Use Committee and complied with the National Institutes of Health guide for the care and use of laboratory animals (NIH Publications No. 8023, revised 1978). All efforts were made to minimize animal suffering and to reduce the number of animals used.

### Experimental Design and Stress Protocol

**Figure 1:**
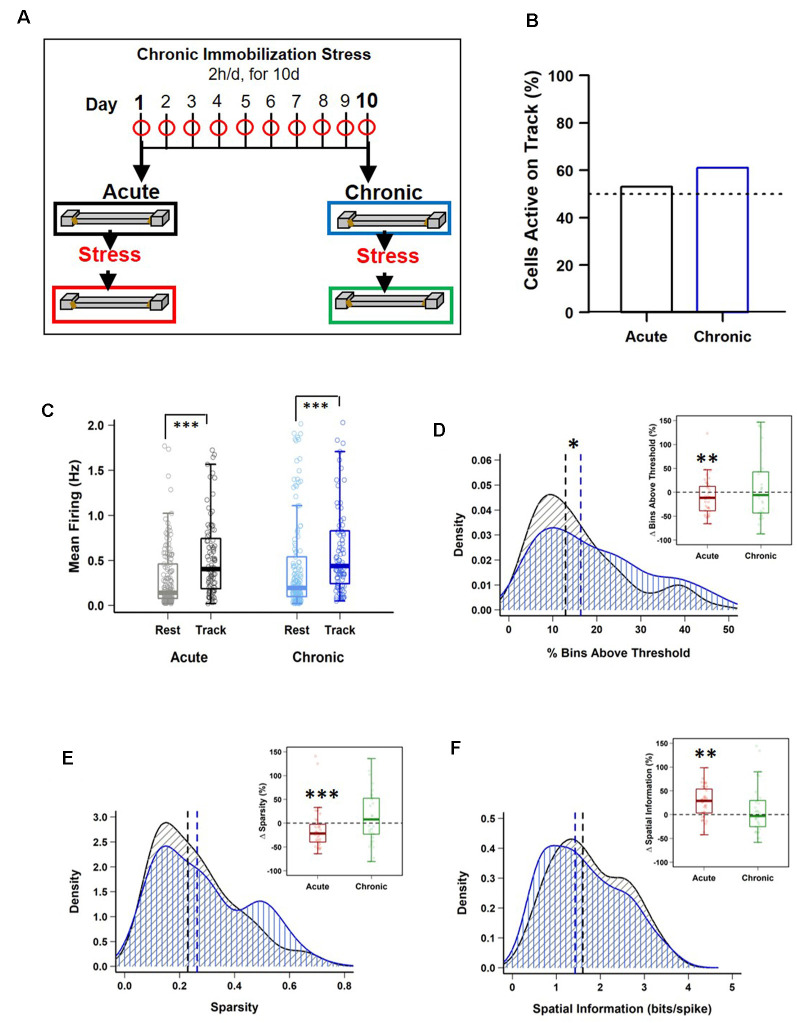
Impact of stress on CA1 place cell activity. **(A)** Schematic representation of the chronic immobilization stress (CIS) protocol and experimental design. **(B)** Percentage of pyramidal cells active during exploration (RUN) compared to quiet wakefulness/sleep (REST) period, before stress administration (Pre-stress), on day-1 (black) and day-10 (blue), [day-1: 95/180 (53%) vs. day-10: 101/166 (61%), *p* = 0.164, chi-square test]. Dotted line represents 50%. **(C)** Pre-stress mean firing rates between REST and RUN on day-1 and day-10 [LMMs: main effect of day, *F*_(1,538)_ = 3.988, *p* = 0.046; main effect of session, *F*_(1,538)_ = 64.02, *p* = 7.708 × 10^−15^; interaction, *F*_(1,538)_ = 0.018, *p* = 0.892; *post hoc* Tukey’s test, day-1: REST (*n* = 180) vs. RUN (*n* = 95), *p* < 0.0001, day-10: REST (*n* = 166) vs. RUN (*n* = 101), *p* < 0.0001]. **(D)** Pre-stress place field size density distribution differs between day-1 and day-10 (PRE-Acute (*n* = 95) vs. PRE-Chronic (*n* = 101), *p* = 0.049, KS-test). However, place cells active during RUN, before and after stress exposure, display a decrease in field size on day-1 (PRE-Acute 13.89 ± 1.46 vs. POST-Acute, 11.25 ± 0.96, *V* = 280, *p* = 0.009, Wilcoxon signed-rank test, *n* = 36) but not on day-10 (PRE-Chronic, 19.71 ± 2.39 vs. POST-Chronic, 17.81 ± 1.99, *V* = 186, *p* = 0.316, Wilcoxon signed-rank test, *n* = 34). **(E)** Pre-stress sparsity of place fields does not differ between day-1 and day-10 (PRE-Acute (*n* = 95) vs. PRE-Chronic (*n* = 101), *p* > 0.05, KS-test). However, place cells active during RUN, before and after stress exposure, display a decrease in sparsity-index on day-1 (PRE-Acute, 0.22 ± 0.02 vs. POST-Acute, 0.18 ± 0.02, *V* = 307, *p* = 1.47 × 10^−4^, Wilcoxon signed-rank test, *n* = 36) but not on day-10 (PRE-Chronic, 0.27 ± 0.03 vs. POST-Chronic, 0.28 ± 0.02, *V* = 145, *p* = 0.90, Wilcoxon signed-rank test, *n* = 34). **(F)** Pre-stress information content (bits/spike) of place fields does not differ between day-1 and day-10 [PRE-Acute (*n* = 95) vs. PRE-Chronic (*n* = 101), *p* > 0.05, KS-test]. However, place cells active during RUN, before and after stress exposure, display a significant increase on day-1 (PRE-Acute, 1.96 ± 0.16 vs. POST-Acute, 2.22 ± 0.14, *V* = 91, *p* = 0.002, Wilcoxon signed-rank test, *n* = 36) but not on day-10 (PRE-Chronic, 1.68 ± 0.17 vs. POST-Chronic, 1.59 ± 0.15, *V* = 167 *p* = 0.643, Wilcoxon signed-rank test, *n* = 34). All box plots represent interquartile range (IQR, 25th–75th percentiles), median is the thick line in the box and whiskers extend to 1.5 times the IQR. The black and red dotted lines on density plots display median values. *p < 0.05, **p < 0.01, ***p < 0.001, *N* = 5 mice.

Mice were habituated to the small sleep-box as well as a linear track daily, and after surgery, mice were again habituated to the sleep-box in which later all “rest” data was collected. Thus, mice were completely habituated to the experimenter, room, sleep box, etc., minimizing the contribution of other (non-stress) repetitive factors/experiences to the changes we observed in the physiology of the hippocampus. Mice underwent the same CIS protocol as described previously (Tomar et al., 2015). Briefly, mice experienced complete immobilization (2 h/day for 10 consecutive days: Figure 1A) in rodent immobilization bags, without access to either food or water. During the actual experiment, all mice experienced a familiar track twice, the first before (PRE) and second after the stress exposure (POST), on the first day (Acute) and the last day (Chronic) of a CIS paradigm thus providing us with four conditions: (i) PRE-Acute; (ii) POST-Acute; (iii) PRE-Chronic; and (iv) POST-Chronic. Each track (RUN) epoch was bracketed by Rest-state (REST) epochs and each epoch was ~ 30 min.

### Surgery, Recordings, and Histology

Mice were anesthetized using Avertin (2,2,2-tribromoethanol; Sigma-Aldrich, 476 mg/kg, i.p.) and were surgically implanted with a microdrive (manufactured with the assistance of the Advanced Manufacturing Support Team, RIKEN Center for Advanced Photonics, Japan). The microdrive housed eight independently movable tetrodes (14 μm diameter, nichrome) and was placed above the right dorsal hippocampus (coordinates from Bregma: AP −1.8 mm; ML + (1.2 mm). Prior to surgery, tetrodes were gold plated to lower impedance down to a range of 100–250 kΩ. Tetrodes were gradually lowered over the course of several days, such that by the start of the experiment they reached the CA1 stratum pyramidale. Data were acquired using a 32-channel Digital Lynx 4S acquisition system (Neuralynx, Bozeman, MT, USA). Signals were sampled at 32,556 Hz and spike waveforms were filtered between 600 Hz and 6 kHz. Skull screws located above the cerebellum served as a ground, and a tetrode positioned in the superficial layers of the neocortex, and devoid of spiking activity, was used for reference. Three to four weeks after surgery, when all tetrodes reached the CA1 stratum pyramidale, evident by multiple large amplitude spikes and SPW-Rs, the experiment was initiated. During REST epochs, the mice were located in a small circular sleep box (15-cm diameter). At the conclusion of the experiment, mice underwent terminal anesthesia (Avertin), and electric current (30 μA, for 8 s) was administered through each electrode to mark their locations. Transcardial perfusion was carried out using saline followed by 4% paraformaldehyde (PFA) followed by a further 24-h fixation in 4% PFA. Brains were sliced using a vibratome (Leica) to prepare coronal slices (50 μm thick) and inspected by standard light microscopy to confirm electrode placement.

### Unit Isolation and Spike Analysis

Spike sorting was performed by an automatic spike sorting program [KlustaKwik (Harris et al., 2000)], followed by manual adjustments of the cluster boundaries using SpikeSort3D software (Neuralynx). Candidate clusters with <0.5% of spikes displaying an inter-spike-interval shorter than 2 ms, a total number of spikes exceeding 50, having a cluster isolation distance value (Schmitzer-Torbert et al., 2005) ≥10, spike halfwidth (peak-to-trough >170 μs and complex spike index (CSI; McHugh et al., 1996) >5 were considered as pyramidal cells and were used for further analysis.

### Place Cell Properties

Pyramidal cells that were active during the period of exploration (RUN) with a speed >2 cm/s on the linear track, had field size ≥6 bins and covered less than 50% of the track were considered place cells. Peak firing rate was defined as the firing rate in the spatial bin containing the maximal value within each firing rate map. The place field size was defined as the number of spatial bins where place cell field firing exceeded 20% of the peak firing rate. The mean firing rate was calculated by dividing the number of spikes which occurred within periods when velocity exceeded 2 cm/s by that period’s duration and then these values were averaged. CSI is defined as CSI = 100 *(pos − neg), where “pos” is the number of inter-spike intervals positively contributing to CSI, that is, preceding spikes with larger amplitudes and following spikes with smaller amplitudes (complex bursts) occurring within 3 ms (refractory period) and 15 ms (maximum inter-spike interval defining a burst); “neg” is the number of inter-spike intervals that contribute negatively to CSI, i.e., violating either or both these rules. A “burst” was defined as at least two spikes occurring within a 10 ms time bin. The burst detection and analysis were performed using MATLAB scripts previously described in Bakkum et al. (2014). Place field “sparsity” was computed as previously described in Resnik et al. (2012). Briefly, “sparsity” was defined as a number ranging from 0 to 1, where 0 corresponds to a firing rate map which consists of equal firing rate values in every visited spatial bin. The firing rate map with sparsity value 1 corresponds to the case when all the spikes generated by any given cell occurred in a single spatial bin. Spatial Information (SI, bits/spike) was calculated as previously reported (Skaggs et al., 1993); Briefly SI = sum{P_spk_(i) * log2 [P_spk_(i)/P_occ_(i)]}, where P_spk_(i) is the probability of spiking in bin “i” and “P_occ_(i)” is the occupancy probability in bin “i”. The “P_spk_” and “P_occ_” values were computed from the rate and occupancy maps respectively.

### Power Spectral Density

The Power Spectral Density (PSD) during exploratory behavior was calculated by using Welch’s averaged modified periodogram method with a 2,048-sample (1.26 s) window size, 50% overlap and 4,096 FFT points (2.52 s) resulting in a time-varying spectrogram. The PSD curves corresponding to time bins when the animal’s velocity was above 6 cm/s were averaged yielding a single PSD curve for each of the four experimental conditions. In order to account for power fluctuations caused by differences in position/impedance of the electrodes and make PSD values comparable across mice, we normalized each PSD curve by its own mean power within the 0–3 Hz band.

### Quantification of Modulation of Firing Rate and Gamma Oscillations by Animal’s Running Speed

Instantaneous running speed curves were obtained by element-wise division of relative changes in the animal’s position between video frames by correspondent inter-frame timestamps. The resulting signal was then smoothed with a 2.5-SD gaussian kernel. For every place cell, all the spikes fired by the cell when the animal was running along the track were binned, using the camera’s frame rate (1/30 s as the bin size, yielding an instantaneous firing rate curve. Instantaneous running speed values were then binned using logarithmically distributed velocity values. The resulting index matrix (second return value of the MATLAB histc() function) was then used to calculate the mean firing rate of the cell within each running speed bin. Modulation of LFP power in gamma frequency bands by the animal’s running speed was assessed by first down-sampling the LFP signal to 400 Hz and up-sampling the previously calculated instantaneous velocity curve using linear interpolation method to the same sampling frequency value as the down-sampled LFP. The resulting LFP signal was then filtered in the target frequency bands (slow and fast gamma) and converted into instantaneous power values by calculating the absolute value of the Hilbert transform of the filtered LFP trace. The up-sampled running speed curve was then binned using the same logarithmically distributed velocity values, and corresponding mean fast and slow gamma power was calculated by using the method described above for each velocity bin.

### Theta/Gamma Phase-Locking to Spikes

The phase relationship between spikes and theta LFP was calculated as previously described (Siapas et al., 2005). Briefly, the instantaneous theta phase was derived from the Hilbert-transformed LFP trace filtered in the theta band (6–12 Hz). Peaks and troughs were assigned 0- and 180° phases respectively, with spike phase calculated using interpolation, a method not sensitive to theta wave asymmetry. The resultant phases were converted to firing probability histograms (10° bin size) while limiting spikes to time periods when the animal’s velocity exceeded 6 cm/s. Significance of the phase locking, preferred firing phase, strength of modulation, and statistical comparison of phase values were calculated using functions from the Circular Statistics Toolbox (Berens, 2009). Gamma/spikes modulation was computed in a similar manner; the calculation was performed using LFP traces filtered in slow gamma (30–50 Hz) and fast gamma (55–90 Hz) frequency bands. Due to the transient nature of gamma oscillations, additional gamma “bursts” detection was performed by calculating time periods when instantaneous power (absolute value of Hilbert transform) of gamma-band filtered LFP trace exceeded various threshold values (in Standard Deviations, 0.5 SD, 1 SD, 2 SD) above mean value of the trace.

### Cross-Frequency Coupling Between Theta and Gamma Oscillations

Cross-Frequency Coupling (CFC) was calculated as described previously (Tort et al., 2010). To reliably detect the phenomena, relatively long chunks of LFP representing a consistent behavior state are necessary, thus time periods when the mouse was running along the track were used in this analysis. LFP data of each lap was first down-sampled to 800 Hz, z-scored and converted to time-varying power over multiple frequency bands matrices by using wavelet transform (mother wavelet function: Morlet, wavelet parameter: 5). Then modulation index (MI) values were calculated for each pair of low (4–20 Hz) and high (30–300 Hz) frequency bands. The significance of the MI values was assessed by using the permutation method (N_perm_ = 200), for details see Tort et al. (2010).

### Statistical Analysis

All statistical analyses were performed in R software (3.3.2). The normality of distributions was not assumed, so comparisons were made using non-parametric statistics. For between-group comparisons, Wilcoxon rank-sum tests were used, while for cells matched between two epochs, Wilcoxon signed-rank tests were used to test the equality of medians. Two-way ANOVAs (aov function, stats package) followed by Tukey’s honestly significant difference (HSD) test (TukeyHSD function, stats package) was used to test for differences between treatments. Overall differences in place cell properties were assessed using linear mixed effects models (LMMs), where mouse identity was specified as a random factor and day and behavior state were specified as fixed factors. The output of the *lmer* function was summarized as an ANOVA table (*anova* function, stats package). Similarly, comparisons for power distributions across various frequency bands in LFP signals were assessed using LMMs, where mouse identity was specified as a random factor and frequency bands as categorical variables were specified as fixed factors. Correlation between parameters was calculated using Pearson’s correlation coefficient analysis (base package). The dependence of a parameter on another was calculated by employing standardized major axis (SMA) regression (*sma* function, smatr package). Comparisons between regression lines were made by likelihood ratio tests (*sma* function, smatr package). For density curve analysis, the Kolmogorov-Smirnov test was employed (*ks.test*, stats package). For phase-locking analysis, statistical analyses were performed on 10° binned data however for visualization purposes data is presented in 30° bins. Boxplots represent Interquartile Range (IQR, 25th–75th percentiles), the median is the thick line housed in the box and whiskers extend to 1.5 times the IQR. No data points were removed as outliers either for making boxplots or for statistical analysis, however for visualization purposes, axes of graphs were readjusted. All statistical tests used were two-sided, and unless otherwise stated, the significance threshold for all tests was set at *p* < 0.05 and *p*-values are shown as follows: *p < 0.05; **p < 0.01; ***p < 0.001.

## Results

The main aim of this study was to examine the differential impact of acute and chronic stress on CA1 spatial coding and hippocampal physiology. To this end, we employed a longitudinal design similar to that employed in previous studies that contrasted neural activity from the same rodents before and after they received exposure to stress (Kim et al., 2007; Ghosh et al., 2013; Tomar et al., 2021). Specifically, we monitored CA1 place cell activity and theta (6–12 Hz) and gamma oscillations (30–90 Hz) during two track exploration sessions: one occurring before (PRE) and second after (POST) the stress exposure, on the first day (Acute) and the last day (Chronic) of a CIS paradigm (Figure 1A; see “Materials and Methods” section), thus providing us with four conditions: (i) PRE-Acute; (ii) POST-Acute; (iii) PRE-Chronic; and (iv) POST-Chronic.

### Differential Impact of Acute and Chronic Stress on Spatial Tuning of CA1 Place Cells

Our recordings from the dorsal CA1 region of the hippocampus (Supplementary Figure 1A) during baseline activity state (REST) yielded a total of 180 pyramidal cells on day-1 (Acute) and 166 pyramidal cells on day-10 (Chronic). No major differences in firing rates was observed, although bursting activity showed a small, but significant, increase at the chronic time point (Table 1). Next, we assessed the impact of stress on mouse behavior during track exploration (RUN) by employing ANOVA statistics where the “main effect of day” signifies the comparisons made between day-1 and day-10 (i.e., after a single exposure and repeated stress) while the “main effect of session” means comparisons made before and after exposure to stress. We observed no discernible change in behavior as the total number of laps traveled by mice did not differ between sessions across days (2-way repeated measures ANOVA: main effect of day, *F*_(1,4)_ = 0.564, *p* = 0.467; main effect of session, *F*_(1,4)_ = 1.459, *p* = 0.250; interaction, *F*_(1,4)_ = 0.001, *p* = 0.974, *N* = 5 mice). Similarly, distance traveled on the track did not differ between days and sessions (2-way repeated measures ANOVA: main effect of day, *F*_(1,4)_ = 1.873, *p* = 0.243; main effect of session, *F*_(1,4)_ = 6.699, *p* = 0.061; interaction, *F*_(1,4)_ = 0.465, *p* = 0.533, *N* = 5 mice). These data demonstrated that neither acute nor chronic stress strongly affected mouse locomotor behavior.

**Table 1:** Pyramidal cell properties during baseline activity and exploratory states on day-1 and day-10.

Parameters	First-day (REST; *n* = 180 cells)	Last-day (REST; *n* = 166 cell)	Statistics
Peak firing (Hz)	4.60 ± 0.69	5.68 ± 0.59	W = 12,556, *p = 0.01
Mean firing (Hz)	0.49 ± 0.07	0.53 ± 0.06	W = 13,316, *p* = 0.08
Complex Spike Index	22.21 ± 0.95	23.48 ± 1.11	W = 14,384, *p* = 0.55
Burst duration	7.72 ± 0.19	7.96 ± 0.22	W = 12,189, *p = 0.0108
Burst ratio	0.32 ± 0.01	0.36 ± 0.01	W = 13,306, *p* = 0.08
Spikes per burst (n)	2.25 ± 0.01	2.32 ± 0.02	W = 12,267, **p = 0.0051
Place cell properties			
	First-day (RUN; *n* = 95 cells)	Last-day (RUN; *n* = 101 cell)	Statistics
Peak firing (Hz)	4.33 ± 0.30	4.94 ± 0.39	W = 4,339, *p* = 0.248
Mean firing (Hz)	0.56 ± 0.06	0.76 ± 0.09	W = 4,289, *p* = 0.201
Complex Spike Index	20.31 ± 1.16	21.39 ± 1.11	W = 4,505, *p* = 0.463
Burst duration	7.40 ± 0.10	7.23 ± 0.16	W = 6,824, *p* = 0.128
Burst ratio	0.34 ± 0.01	0.36 ± 0.01	W = 5,870, *p* = 0.477
Spikes per burst (n)	2.26 ± 0.02	2.27 ± 0.02	W = 5,869, *p* = 0.546

Impact of stress on CA1 pyramidal cells during baseline activity (REST) and exploration (RUN). *Top*: comparisons of spiking and bursting properties of pyramidal cells during quiet wakefulness/sleep (REST) between day-1 and day-10. *Bottom*: comparisons of spiking and bursting properties of pyramidal “place” cells during pre-stress track exploration (RUN) between day-1 and day-10. Data is presented as Mean ± SEM, Wilcoxon rank-sum test, *p < 0.05, **p < 0.01, *N* = 5 mice.

Next, we examined the impact of stress on the location-specific activation of CA1 pyramidal “place” cells during linear track exploration (O’Keefe and Dostrovsky, 1971). The fraction of neurons that passed place cell criteria during RUN (see “Materials and Methods” section) was similar on the first and the last day of CIS (Figure 1B; day-1: 95/180 (53%) vs. day-10: 101/166 (61%), *p* = 0.164, *χ*^2^ test, *N* = 5 mice), indicating that chronic stress did not alter activation of place cell ensembles. As expected, the mean firing of cells was significantly higher during RUN compared to the baseline REST session (Figure 1C), with no discernible effect of repeated stress exposure (averaged firing; LMMs: main effect of day, *F*_(1,538)_ = 3.988, *p* = 0.046; main effect of session, *F*_(1,538)_ = 64.02, *p* = 7.708 × 10^−15^; interaction, *F*_(1,538)_ = 0.018, *p* = 0.892, *n* = 542 cells, *N* = 5 mice). Thus, pyramidal cells increased their discharge rate during spatial coding and neither acute nor chronic stress affected this property of pyramidal cells.

We then performed detailed analysis of place cell properties. Place field size, defined as the proportion of the track that a place cell was active on, showed a main effect of day (Figure 1D; size; LMMs: main effect of day, *F*_(1,369)_ = 8.660, *p* = 0.0035; main effect of session, *F*_(1,369)_ = 1.201, *p* = 0.274; interaction, *F*_(1,369)_ = 0.019, *p* = 0.890, *n* = 373 cells, *N* = 5 mice). Further, density distribution of field size during PRE-stress sessions changed after repeated stress such that compared to day-1, on day-10, a greater fraction of cells had larger place fields [day-1 (*n* = 95 cells) vs. day-10, (*n* = 101 cells); KS-test, *p* = 0.049, *N* = 5 mice]. Moreover, neurons, that were active during both PRE and POST stress sessions, displayed a decrease in field size after stress exposure on day-1 (PRE-Acute, 13.89 ± 1.46 vs. POST-Acute, 11.25 ± 0.96, *p* = 0.009, Wilcoxon signed-rank test, *n* = 36 cells, *N* = 5 mice) but not on day-10 (PRE-Chronic, 19.71 ± 2.39 vs. POST-Chronic, 17.81 ± 1.99, *p* = 0.316, Wilcoxon signed-rank test, *n* = 34 cells, *N* = 5 mice). Thus, place fields decreased in size after the acute stress, but expanded after repeated exposure to stress.

Altered place field size alone fails to capture all changes in spatial coding, as previous studies have reported that bigger place fields can be suggestive of both improved spatial coding (Hussaini et al., 2011) and a loss of spatial specificity (McHugh et al., 1996). Thus, we next assessed the impact of stress on spatial tuning by measuring the sparsity-index, a metric of spatial selectivity (Jung et al., 1994). The sparsity-index of individual place cells was also impacted by stress (Figure 1E; sparsity; LMMs: main effect of day, *F*_(1,369)_ = 8.931, *p* = 0.003; main effect of session, *F*_(1,369)_ = 1.929, *p* = 0.166; interaction, *F*_(1,369)_ = 2.017, *p* = 0.156, *n* = 373 cells, *N* = 5 mice). A further analysis of cells that were active before and after exposure to stress confirmed this result, as significantly lower sparsity-index was noticed after acute stress (PRE-Acute, 0.22 ± 0.02 vs. POST-Acute, 0.18 ± 0.02, *p* = 1.47 × 10^−4^, Wilcoxon signed-rank test, *n* = 36 cells, *N* = 5 mice), but not after repeated stress (PRE-Chronic, 0.27 ± 0.03 vs. POST-Chronic, 0.28 ± 0.02, *p* = 0.90, Wilcoxon signed-rank test, *n* = 34 cells, *N* = 5 mice). Further, spatial information content (bits/spike), a parameter which quantifies how much information about the mouse’s location is contained within the activity of a place cell (Skaggs et al., 1993), was also impacted by stress (Figure 1F; information; LMMs: main effect of day, *F*_(1,369)_ = 10.969, *p* = 0.001; main effect of session, *F*_(1,369)_ = 2.586, *p* = 0.109; interaction, *F*_(1,369)_ = 5.414, *p* = 0.0205, *n* = 373 cells, *N* = 5 mice). This was further confirmed as place cells active on the track before and after the exposure to stress also showed a significant increase in information content on day-1 (PRE-Acute, 1.96 ± 0.16 vs. POST-Acute, 2.22 ± 0.14, *p* = 0.002, Wilcoxon signed-rank test, *n* = 36 cells, *N* = 5 mice) but not on day-10 (PRE-Chronic, 1.68 ± 0.17 vs. POST-Chronic, 1.59 ± 0.15, *p* = 0.643, Wilcoxon signed-rank test, *n* = 34 cells, *N* = 5 mice) of the CIS protocol.

Sharpening of place coding after acute stress was not caused by altered firing rate. However, a main effect of day on firing rate was observed (mean firing; LMMs: main effect of day, *F*_(1,369)_ = 13.014, *p* = 3.518 × 10^−4^; main effect of session, *F*_(1,369)_ = 0.381, *p* = 0.537; interaction, *F*_(1,369)_ = 1.362, *p* = 0.244, *n* = 373 cells, *N* = 5 mice, Tukey’s* post hoc*, POST-Acute vs. POST-Chronic, *p* = 0.005). In view of reports that firing rate increases along with running speed of freely behaving rodents (McNaughton et al., 1983), we next asked if differential impact of acute and chronic stress on place coding was result of difference in exploration speed across days or session but found no significant difference (2-way repeated measures ANOVA: main effect of day, *F*_(1,4)_ = 3.106, *p* = 0.103; main effect of session, *F*_(1,4)_ = 1.318, *p* = 0.273; interaction, *F*_(1,4)_ = 0.08, *p* = 0.782, *N* = 5 mice). Further, when we compared firing rate of each place cell across different speed bins (Supplementary Figure 1B) by using speed as a repeated variable and each recording session as non-repeated variable, we noticed a clear pattern of increase in firing rate as the speed increased [2-way mixed ANOVA: main effect of speed, *F*_(1,369)_ = 1,424.752, *p* < 2.22 × 10^−16^, main effect of group, *F*_(3,369)_ = 16.666, *p* = 3.546 × 10^−10^; interaction, *F*_(3,369)_ = 37.866, *p* < 2.22 × 10^−16^, *N* = 5 mice; PRE-Acute (*n* = 95) cells vs. POST-Acute (*n* = 89 cells), *p* = 0.012, PRE-Chronic (*n* = 101 cells) vs. POST-Chronic (*n* = 88 cells), *p* < 0.0001, PRE-Acute (*n* = 95 cells) vs. PRE-Chronic (*n* = 101 cells), *p* = 0.003, Tukey’s* post hoc* test]. Overall, the above data indicate that smaller place fields and enhanced spatial tuning, following acute stress, was not caused by altered speed or relationship between firing rate and speed. However, same was not true for chronic stress.

### Differential Impact of Acute and Chronic Stress on Exploration-Associated Theta and Gamma Oscillations

**Figure 2:**
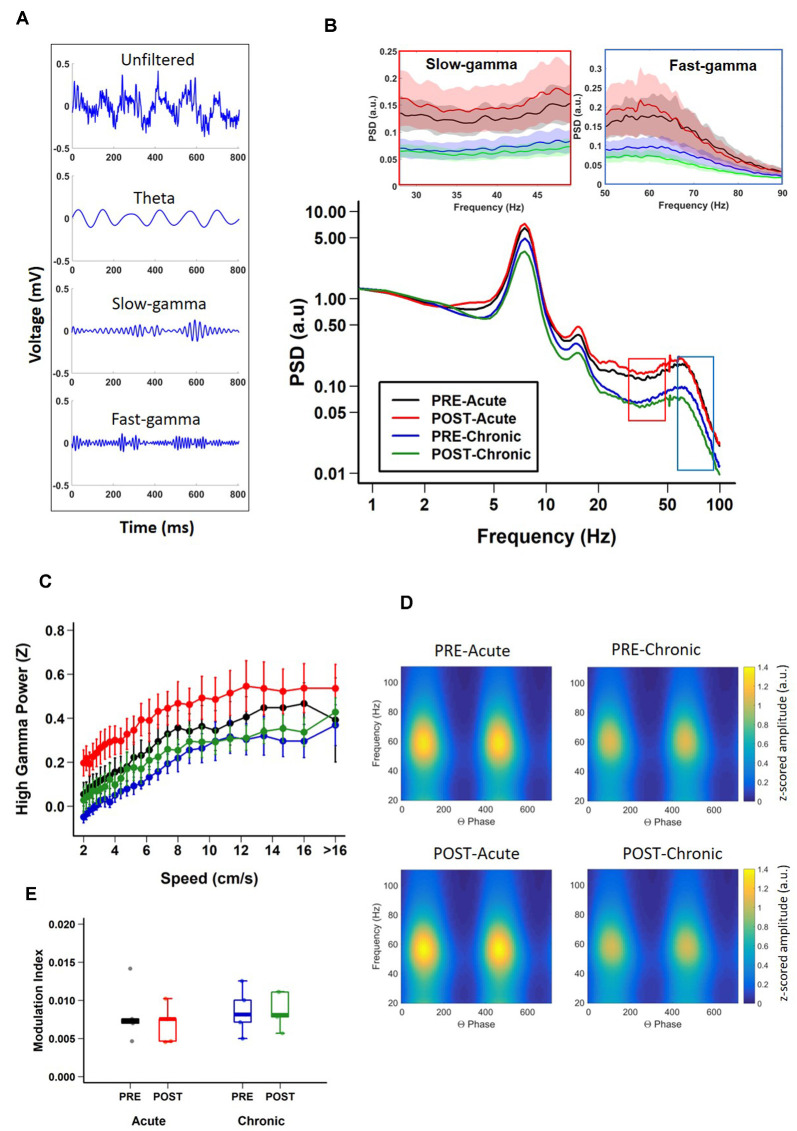
Impact of stress on CA1 oscillatory activity.** (A)** Representative examples of unfiltered (top) and filtered (bottom) local field potentials (LFPs) during track exploration for theta, slow-gamma, and fast-gamma. **(B)** Power spectral density (PSD) curves of CA1 LFPs during linear track exploration (RUN) show no significant differences for theta (6–12 Hz; Theta, 2-way repeated measure ANOVA: day, main effect of day *F*_(1,19)_ = 2.7018, *p* = 0.1262; main effect of session, *F*_(1,19)_ = 0.0427, *p* = 0.8398; interaction, *F*_(1,19)_ = 0.4823, *p* = 0.501). Fast-gamma (55–90 Hz) showed an effect of day but not of session (FG, *right inset*; 2-way repeated measure ANOVA: main effect of day, *F*_(1,19)_ = 6.7062, *p* = 0.0237; main effect of session, *F*_(1,19)_ = 0.1047, *p* = 0.752 interaction, *F*_(1,19)_ = 0.2074, *p* = 0.657). Slow-gamma (30–50 Hz) showed an effect of day but not of session (SG, *left inset*, 2-way repeated measure ANOVA: main effect of day, *F*_(1,19)_ = 8.3668, *p* = 0.0135; main effect of session, *F*_(1,19)_ = 0.0634, *p* = 0.805; interaction, *F*_(1,19)_ = 0.3754, *p* = 0.551). **(C)** Relationship between FG power and running speed on the track (FG; 2-way repeated measure ANOVA : main effect of speed, *F*_(1,36)_ = 471.385, *p* < 2.22 × 10^−16^, main effect of group, *F*_(3,36)_ = 5.306, *p* = 0.015; interaction, *F*_(3,36)_ = 10.033, *p* = 2.261 × 10^−6^, *N* = 5 mice). **(D)** Representative examples of modulation of gamma amplitude by theta phase in dorsal CA1 pyramidal cell layer before (top) and after (bottom) stress exposure on day-1 (left) and day-10 (right). The colorbar represents the z-scored gamma power in arbitrary units (a.u.) for both left and right-side graphs. **(E)** Theta-FG phase-amplitude coupling (top) did not differ across days and sessions (2-way repeated measures ANOVA: day, main effect of day, *F*_(1,4)_ = 0.839, *p* = 0.411; main effect of session, *F*_(1,4)_ = 3.399, *p* = 0.139; interaction, *F*_(1,4)_ = 4.953, *p* = 0.09). Similarly, theta-SG coupling (bottom) was not affected by either acute or chronic stress (2-way repeated measures ANOVA: day, main effect of day, *F*_(1,4)_ = 0.592, *p* = 0.484; main effect of session, *F*_(1,4)_ = 1.756, *p* = 0.256; interaction, *F*_(1,4)_ = 1.02, *p* = 0.370). *N* = 5 mice.

Having established the differential impact of acute and chronic stress on place cell activity, next we asked if the same was true for hippocampal LFPs (Figure 2A) which provides a measure of average synaptic input to a local region (Buzsáki et al., 2012) and to some extent also reflects slow dynamics of spiking in a local region (Rasch et al., 2008). During exploratory behavior, the hippocampal LFP is dominated by prominent large-amplitude theta (6–12 Hz) oscillations (Vanderwolf, 1969; O’Keefe and Dostrovsky, 1971), which play a crucial role in the temporal organization of hippocampal activity (Buzsáki and Moser, 2013). Thus, we examined the impact of acute and chronic stress on theta oscillations. A comparison of the PSD of the LFPs, across sessions (Figure 2B) revealed that theta oscillations were robustly present and power in the theta band was not affected by either acute or chronic stress, as no effect of day or session was observed (theta; 2-way repeated measures ANOVA: day, main effect of day *F*_(1,19)_ = 2.7018, *p* = 0.1262; main effect of session, *F*_(1,19)_ = 0.0427, *p* = 0.8398; interaction, *F*_(1,19)_ = 0.4823, *p* = 0.501, *N* = 5 mice).

In addition to theta, the hippocampus displays occasional low-amplitude, high frequency gamma (30–90 Hz) oscillations (Bragin et al., 1995; Buzsáki et al., 2003; Colgin, 2016). Gamma oscillations consist of distinct subtypes with non-overlapping frequency ranges, slow (SG: 30–50 Hz) and fast (FG: 55–90 Hz) gamma (Schomburg et al., 2014; Colgin, 2016; Middleton and McHugh, 2016; Alexander et al., 2018), and enhanced gamma oscillatory activity has been suggested to reflect dynamic changes in excitatory input into CA1 (Buzsáki and Moser, 2013; Fries, 2015). Thus, we next assessed the impact of stress on these individual gamma bands. Significant decreases in fast-gamma power were evident on day-10 (Figure 2B; FG; 2-way repeated measures ANOVA: main effect of day, *F*_(1,19)_ = 6.7062, *p* = 0.0237; main effect of session, *F*_(1,19)_ = 0.1047, *p* = 0.752; interaction, *F*_(1,19)_ = 0.2074, *p* = 0.657, *N* = 5 mice). Similarly, chronic stress also led to similar decreases in slow-gamma power (Figure 2B; SG; 2-way repeated measures ANOVA: main effect of day, *F*_(1,19)_ = 8.3668, *p* = 0.0135; main effect of session, *F*_(1,19)_ = 0.0634, *p* = 0.805; interaction, *F*_(1,19)_ = 0.3754, *p* = 0.551, *N* = 5 mice). In agreement with a previous report (Chen et al., 2011), the power of gamma oscillations consistently increased across the range of speeds for FG (Figure 2C; 2-way repeated measure ANOVA: main effect of speed, *F*_(1,36)_ = 471.385, *p* < 2.22 × 10^−16^, main effect of group, *F*_(3,36)_ = 5.306, *p* = 0.015; interaction, *F*_(3,36)_ = 10.033, *p* = 2.261 × 10^−6^, *N* = 5 mice). However, the increase in slow gamma power as the speed increased was subtler (SG; 2-way repeated measure ANOVA: main effect of speed, *F*_(1,36)_ = 42.562, *p* = 1.485 × 10^−9^, main effect of group, *F*_(3,36)_ = 5.774, *p* = 0.011; interaction, *F*_(3,36)_ = 5.124, *p* = 0.002, *N* = 5 mice).

The amplitude of gamma oscillations has also been shown to be modulated by the phase of slower underlying theta rhythm (Bragin et al., 1995; Chrobak and Buzsáki, 1998; Canolty et al., 2006) and this theta-phase gamma-amplitude coupling has been suggested to reflect local information processing in hippocampal circuits (Tort et al., 2009; Buzsáki and Wang, 2012). Thus, we next determined the impact of stress on theta-gamma coupling during periods when mice ran along the linear track linear (i.e., when prominent theta oscillations are known to be present) by calculating modulation index (MI), a measure of the strength of coupling between gamma-amplitude and theta phase (Tort et al., 2010). We found no changes in the strength of theta-gamma coupling (Figures 2D,E; theta-gamma; 2-way repeated measures ANOVA: day, main effect of day, *F*_(1,4)_ = 0.839, *p* = 0.411; main effect of session, *F*_(1,4)_ = 3.399, *p* = 0.139; interaction, *F*_(1,4)_ = 4.953, *p* = 0.09, *N* = 5 mice). Further no significant difference was observed either between theta-fast gamma coupling (theta-FG; 2-way repeated measures ANOVA: day, main effect of day, *F*_(1,4)_ = 0.839, *p* = 0.411; main effect of session, *F*_(1,4)_ = 3.399, *p* = 0.139; interaction, *F*_(1,4)_ = 4.953, *p* = 0.09, *N* = 5 mice) or theta-slow gamma coupling (theta-SG; 2-way repeated measures ANOVA: day, main effect of day, *F*_(1,4)_ = 0.592, *p* = 0.484; main effect of session, *F*_(1,4)_ = 1.756, *p* = 0.256; interaction, *F*_(1,4)_ = 1.02, *p* = 0.370, *N* = 5 mice).

Thus, LFP power analysis indicated that while the first exposure to stress did not alter theta and gamma oscillatory activity, repeated stress led to suppression of SG and FG power, but had no impact on CFC between theta and gamma.

### Impact of Acute and Chronic Stress on Temporal Coding (LFP-Spike Interactions)

**Figure 3:**
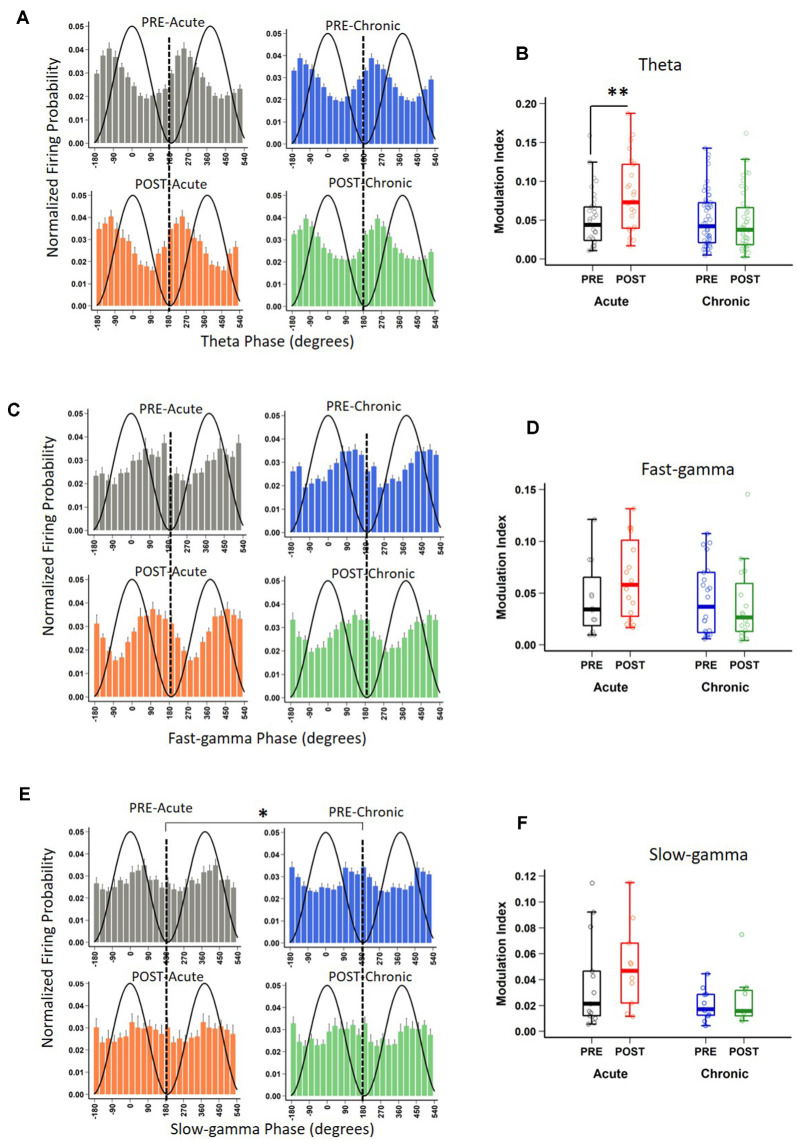
Impact of stress on phase-locking properties of CA1 place cells to theta and gamma oscillations. **(A)** The spiking probability plotted as a function of the phase of theta for significantly theta-modulated place cell populations (Rayleigh *p* < 0.05). Population spiking probability is elevated around the trough and ascending phase of theta (0/360° set for theta peak, 180° for theta trough). **(B)** The strength of theta-phase locking (Modulation index, MI) is altered by stress [LMMs: main effect of day, *F*_(1,155)_ = 1.892, *p* = 0.171; main effect of session, *F*_(1,155)_ = 5.425, *p* = 0.022; interaction, *F*_(1,155)_ = 5.702, *p* = 0.018); *post hoc* Tukey’s test, PRE-Acute (*n* = 35) vs. POST-Acute (*n* = 26), *p* = 0.006, POST-Acute (*n* = 26) vs. POST-Chronic (*n* = 45), *p* = 0.038. **(C)** The spiking probability plotted as a function of the phase of fast-gamma (FG) for significantly FG-modulated place cell populations (Rayleigh *p* < 0.05) is elevated around the trough and descending phase of FG (0/360° set for FG peak, 180° for FG trough) but stress did not affect this phase relationship: (FG, Circular ANOVA, *F*_(3,62)_ = 0.919, *p* = 0.437). **(D)** The strength of FG phase-locking (MI) remains unaltered by stress (FG; LMMs: main effect of day, *F*_(1,62)_ = 0.986, *p* = 0.325; main effect of session, *F*_(1,62)_ = 0.801, *p* = 0.374; interaction, *F*_(1,62)_ = 1.936, *p* = 0.169).** (E)** The spiking probability plotted as a function of the phase of slow-gamma (SG) for significantly SG-modulated place cell populations (Rayleigh *p* < 0.05). Population spiking probability is elevated around the descending phase of SG (0/360° set for SG peak, 180° for SG trough). **(F)** The strength of SG-phase locking (MI) was not significantly altered by stress (SG; LMMs: main effect of day, *F*_(1,37)_ = 3.747, *p* = 0.063; main effect of session, *F*_(1,37)_ = 1.063, *p* = 0.309; interaction, *F*_(1,37)_ = 0.115, *p* = 0.737). Boxplots represent interquartile range (IQR, 25th–75th percentiles), median is the thick line in the box and whiskers extend to 1.5 times the IQR., *p < 0.05, **p < 0.01, *N* = 5 mice.

In addition to rate coding (location-specific spiking), place cells also display temporal coding, reflecting their preference for spiking at specific phases of the concurrent oscillations (O’Keefe, 1976; Fox et al., 1986; Csicsvari et al., 1999). It has been hypothesized that temporal coding supports transient activation of place cell ensembles, a phenomenon central to spatial information processing (Harris et al., 2003; O’Keefe and Burgess, 2005; Buzsáki, 2010; Lever et al., 2014). Knowing that acute and chronic stress differentially alter the place cell rate code, we next asked if they differentially impact temporal coding by assessing the strength and phase preference of CA1 place cell spiking to theta and gamma oscillations. Similar to a previous report (Jones and Wilson, 2005), the majority (66–73%) of CA1 place cells demonstrated significant modulation by theta (Rayleigh test of uniformity *p* < 0.05) and neither acute nor chronic stress affected this distribution (Table 2; *p* = 0.814, *χ*^2^ test). Further, as expected based on earlier studies (Csicsvari et al., 1999; Jones and Wilson, 2005; Jadhav et al., 2016), the majority of neurons displayed a preference to spike near the trough of the theta oscillation (Figure 3A) and this mean preferred phase for theta-modulated cells was not affected by stress (Supplementary Figure 1C; phase; Circular ANOVA, *F*_(3,155)_ = 1.305, *p* = 0.274, *n* = 159 cells, *N* = 5 mice). Interestingly, however, the strength of theta-phase locking (Figure 3B) was significantly increased specifically after acute stress (MI; LMMs: main effect of day, *F*_(1,155)_ = 1.892, *p* = 0.171; main effect of session, *F*_(1,155)_ = 5.425, *p* = 0.022; interaction, *F*_(1,155)_ = 5.702, *p* = 0.018, *n* = 159 cells, *N* = 5 mice); *post hoc* Tukey’s test, PRE-Acute (*n* = 35 cells) vs. POST-Acute (*n* = 26 cells), *p* = 0.006, POST-Acute (*n* = 26 cells) vs. POST-Chronic (*n* = 45 cells), *p* = 0.038.

**Table 2:** Distribution of place cells phase-locked to theta and gamma oscillations on day-1 and day-10.

Rhythm	PRE-Acute	POST-Acute	PRE-Chronic	POST-Chronic	Statistics
Theta	35/48 (73%)	26/38 (68%)	53/73 (73%)	45/68 (66%)	*p* = 0.814, *χ*^2^ test
Fast-gamma	30/66 (45%)	26/63 (41%)	34/78 (44%)	30/74 (40%)	*p* = 0.935, *χ*^2^ test
Slow-gamma	27/74 (36%)	30/87 (34%)	29/91 (32%)	31/87 (36%)	*p* = 0.928, *χ*^2^ test

Similar to the modulation of spiking by theta, the precise timing of pyramidal cell firing can also be entrained by gamma oscillations (Csicsvari et al., 2003). Thus, we next performed the spike phase-locking analysis of gamma oscillations during high velocity periods (speed > 6 cm/s) on the track. A large fraction (40–45%; Rayleigh test of uniformity *p* < 0.05) of CA1 place cell population displayed a significant phase preference during FG and neither acute nor chronic stress affected this distribution (Table 2; *p* = 0.935, *χ*^2^ test). Moreover, stress did not alter the preferred phase (phase; Circular ANOVA, *F*_(3,108)_ = 1.932, *p* = 0.129, *n* = 112 cells, *N* = 5 mice, Supplementary Figure 1D) or strength (MI; LMMs: main effect of day, *F*_(1,108)_ = 1.499, *p* = 0.224; main effect of session, *F*_(1,108)_ = 1.292, *p* = 0.258; interaction, *F*_(1,108)_ = 0.055, *p* = 0.815, *n* = 112 cells, *N* = 5 mice) of the phase-locking of CA1 pyramidal cells. As gamma oscillations are more transient than theta during locomotion, we next focused on periods of strong FG on the track regardless of animal’s speed or position on the track (see “Materials and Methods” section), and again observed that stress did not alter either the preferred phase (Figure 3C; phase: Circular ANOVA, *F*_(3,62)_ = 0.919, *p* = 0.437, *n* = 66 cells, *N* = 5 mice) or the strength of FG phase-locking (Figure 3D; MI: LMMs: main effect of day, *F*_(1,62)_ = 0.986, *p* = 0.325; main effect of session, *F*_(1,62)_ = 0.801, *p* = 0.374; interaction, *F*_(1,62)_ = 1.936, *p* = 0.169, *n* = 66 cells, *N* = 5 mice).

Unlike FG, the preferred phase of the cells modulated by SG was more variable across the population during high velocity periods on the track. The proportion of CA1 place cells with a significant SG phase preference was ~32–36%; (Rayleigh test of uniformity *p* < 0.05) and showed no differences across four sessions (Table 2; *p* = 0.928, *χ*^2^ test). Nonetheless, following chronic stress we did observe a small, yet significant change in the mean preferred phase (Supplementary Figure 1E; phase: Circular ANOVA, *F*_(3,113)_ = 6.862, *p* = 2.74 × 10^−4^, *n* = 117 cells, *N* = 5 mice), but noticed no change in the strength of modulation (MI: LMMs: main effect of day, *F*_(1,113)_ = 0.182, *p* = 0.671; main effect of session, 0.035, *p* = 0.852; interaction, *F*_(1,113)_ = 2.358, *p* = 0.127, *n* = 117 cells, *N* = 5 mice). Finally, when we focused on the phase-locking of place cells specifically during periods of strong SG regardless of the animal’s speed or position on the track, we found that chronic stress led place cells to fire at the later phase of SG (Figure 3E-phase; Circular ANOVA, *F*_(3,37)_ = 5.057, *p* = 0.005, *n* = 41 cells, *N* = 5 mice; *post hoc* Watson-Wheeler test, PRE-Acute (*n* = 13 cells) vs. PRE-Chronic (*n* = 11 cells), *p* = 0.036). Chronic stress showed a trend that it affected the strength of SG-phase locking but it was not significant (Figure 3F; MI: LMMs: main effect of day, *F*_(1,37)_ = 3.747, *p* = 0.063; main effect of session, *F*_(1,37)_ = 1.063, *p* = 0.309; interaction, *F*_(1,37)_ = 0.115, *p* = 0.737, *n* = 41 cells, *N* = 5 mice).

Thus, while first exposure to stress increased the strength of theta phase-locking demonstrating the facilitatory effects of acute stress on temporal coding, chronic stress disrupted temporal coding as the mean phase and the strength of phase-locking of place cells to slow-gamma oscillations was altered on day-10.

## Discussion

Despite reports that acute stress positively impacts cognition, including hippocampal information processing (Henckens et al., 2009; Yuen et al., 2009; Kirby et al., 2013), it is not yet clear how this is reflected in hippocampal place cell activity and LFP-spike interactions, two neural processes involved in spatial coding (O’Keefe and Dostrovsky, 1971; Buzsáki, 2010; Lever et al., 2014). Here, we show that while after the first exposure to stress (Acute stress) or the last exposure to stress (Chronic stress) the averaged speed and distance covered on the track were not affected. However, after acute stress, CA1 place cells displayed refined spatial coding (Figure 1D), increased information content (Figure 1F) and decreased sparsity-index (Figure 1E). Further, chronic, but not acute stress, led to decreased LFP power in the slow-gamma (SG; 30–50 Hz) and fast-gamma (FG 55–90 Hz) bands (Figure 2B) along with an increase in place field size. Furthermore, the strength of theta phase-locking to CA1 place cells increased after acute stress (Figures 3A,B), however, the mean phase of slow-gamma phase-locking was altered as stress became chronic (Figure 3E). Together, these results indicate that acute stress has a facilitatory impact on hippocampal information coding, while chronic stress impairs it.

Stress impacts on hippocampal functionality have been hypothesized to follow a U-shaped curve, where exposure to acute stress facilitates, while chronic stress disrupts, hippocampal function (Salehi et al., 2010; McEwen et al., 2016). Our results of enhanced spatial information content and increased strength of phase-locking after acute stress, as well as broader place fields and suppressed gamma power after repeated stress are consistent with stress exerting a U-shaped impact on hippocampal function in the intact brain. Rate and temporal coding of CA1 pyramidal cells aid spatial information processing (O’Keefe, 1976; O’Keefe and Recce, 1993; O’Keefe and Burgess, 2005). Since acute stress facilitated both types of coding (i.e., improved spatial tuning and strength of theta phase-locking), the idea that acute stress effects on hippocampal spatial coding are indeed facilitatory in nature is not far-fetched. Mechanistically, the facilitatory effects of acute stress on hippocampal coding are likely brought about by the combined action of a cocktail of neuromodulators released by stress-induced activation of sympatho-adrenal medullary (SAM)-pathways (Cadle and Zoladz, 2015; Gunn and Baram, 2017). Future studies are needed to further investigate the role of SAM-activated neuromodulation on CA1 spatial coding.

Instantaneous coupling between theta and gamma oscillations in hippocampal networks is thought to represent dynamic processing in hippocampal circuits (Buzsáki and Wang, 2012). A previous study using evoked auditory potentials also noted a decrease in gamma power following CIS and concluded that chronic stress disrupts functional connectivity within the hippocampal circuitry (Ghosh et al., 2013). The same conclusion was also reached by Passecker et al. (2011) who studied the impact of repeated exposure to photic stress on hippocampal spatial coding. Gamma oscillations route information flow in hippocampal circuits including slow CA1 gamma which reflects interactions between CA3-CA1 neuronal networks (Montgomery and Buzsáki, 2007), while fast CA1 gamma indicates CA1-MEC interactions (Colgin et al., 2009; Colgin, 2016). Our observation of decreased slow and fast gamma power following chronic, but not acute stress reflects the poor functional connectivity in hippocampal-entorhinal circuits in chronically stressed subjects. Importantly, functional connectivity was not altered after acute stress, as place maps were more informative of animal’s location in space.

What factors may lead to weakened functional connectivity in hippocampal circuits in response to repeated stress? Earlier studies have reported that chronic, but not acute stress, causes dendritic shortening and debranching and synaptic loss on apical branches of pyramidal cells in areas CA3 and CA1 (Magariños and McEwen, 1995; Conrad et al., 1999; Sousa et al., 2000; Sandi et al., 2003). Hitherto, the functional consequences of these structural changes have not been well understood. Since apical dendritic branches of CA1 pyramidal cells are the loci of Schaffer collateral inputs (from CA3) and temporoammonic pathways (from the medial entorhinal cortex; MEC), chronic stress-induced CA1 dendritic shrinkage likely reflects poor information flow into CA1 circuits (Colgin et al., 2009). Knowing that CA1 SG oscillations reflect interactions between CA1 and CA3/CA2 circuitry (Colgin et al., 2009; Middleton and McHugh, 2016; Alexander et al., 2018), while FG represents the interactions between area CA1 and medial entorhinal cortical circuits (Colgin et al., 2009; Kemere et al., 2013), it is not surprising that chronic (but not acute stress) causes a decrease in SG and FG power. In addition, AMPA-dependent synaptic plasticity is implicated in modulating gamma phase-locking of pyramidal cells by altering inhibitory-excitatory balance in area CA1 (Kitanishi et al., 2015). Knowing that chronic stress alters hippocampal synaptic plasticity (Alfarez et al., 2003) and AMPA-dependent synaptic transmission in the temporoammonic-CA1 pathway (Kallarackal et al., 2013), it is likely that chronic stress-induced altered synaptic plasticity is another potential candidate underlying chronic stress phenotypes noticed in this study. Further, inhibitory neuronal activity plays a key role in the generation of gamma oscillations, as well as the phase-locking of pyramidal cells to gamma oscillations (Bartos et al., 2002; Buzsáki and Wang, 2012). Reports that chronic stress causes decreases in hippocampal PV^+^ inhibitory neuronal density by ~20–25% (Zaletel et al., 2016; Csabai et al., 2017) suggests that decreased gamma power and altered gamma phase-locking of CA1 place cells, observed in this study, are contributed by CIS-induced weakening of inhibition. Future studies will have to assess the differential contributions of chronic stress-induced altered inhibition, synaptic plasticity and dendritic atrophy to altered place cell activity, gamma oscillations and phase-locking phenotypes observed in this study.

Inescapability along with repeatability are two key components of modern-day life stress. Therefore, the majority of animal models of chronic stress have inescapability and repeatability built into them (Chattarji et al., 2015). The immobilization stress (and the closely related restraint stress) models are particularly popular in experimental stress neurobiology research as in addition to psychological stress (involving inescapability and repeatability aspects), these stress models also exert physical stress on the subject (McEwen, 1999). Since this study only employed immobilization stress, it is not yet clear if the changes observed in this study would be elicited by other models of stress. Thus, future studies employing two or more different animal models of chronic stress are needed to clarify if only the immobilization-related physical model of stress or any stress could differentially alter spatial coding and gamma oscillations when applied either once or repeatedly.

A decrease in gamma (30–90 Hz) power and broadening of place field size after repeated stress exposure indicates that acute and chronic stress differentially alter information coding in the CA1 subregion. In view of reports that hippocampal phase-locking is altered in neurodegenerative disease models (Booth et al., 2016; Mably et al., 2017), for which stress is a risk factor (Bisht et al., 2018), it is not surprising that we observed altered phase-locking in response to CIS. These data further add to accumulating evidence that repeated stress negatively impacts spatial coding (Kim et al., 2007; Chattarji et al., 2015; Tomar et al., 2015). Spike-LFP interactions are responsible for not only local computations within a circuit but also coordinate activity across distant but connected circuits (Buzsáki and Freeman, 2015; Harris and Gordon, 2015; Colgin, 2016; Shin and Jadhav, 2016; Makino et al., 2019). Thus, our results of altered oscillatory and place cell activity have implications for neural computations across various memory-related circuits connected to the hippocampus.

In conclusion, our results of acute stress-induced increased information content of place cells and strengthening of phase-locking to theta oscillations further support the idea that acute stress facilitates hippocampal neural computations. Based on these findings, we propose that acute and chronic stress differentially, likely opposingly, influence hippocampal information processing.

## Data Availability Statement

The raw data supporting the conclusions of this article will be made available by the authors, without undue reservation.

## Ethics Statement

The animal study was reviewed and approved by RIKEN Institutional Animal Care and Use Committee.

## Author Contributions

AT and TM conceived the study. AT performed all experiments. AT, DP, and TM analyzed the data. AT and TM wrote the manuscript with inputs from DP. Funding provided by TM. All authors contributed to the article and approved the submitted version.

## Conflict of Interest

The authors declare that the research was conducted in the absence of any commercial or financial relationships that could be construed as a potential conflict of interest.

## Supplementary Material

SUPPLEMENTARY FIGURE 1 The impact of stress on the relationship between CA1 place cell spiking vs. running speed and LFP oscillatory phase. ** (A)** Coronal section of the hippocampus showing the tetrode locations (black arrows) at the CA1 pyramidal layer. **(B)** Dependence of firing rate on the speed was affected by stress (2-way mixed ANOVA: main effect of speed, *F*_(1,370)_ = 1,424.752, *p* < 2.22 × 10^−16^, main effect of group, *F*_(3,370)_ = 16.666, *p* = 3.546 × 10^−10^; interaction, *F*_(3,370)_ = 37.866, *p* < 2.22 × 10^−16^, *N* = 5 mice). On day-1, after acute stress, place cells displayed lower firing over the range of speed bins examined (PRE-Acute (*n* = 95) cells vs. POST-Acute (*n* = 89 cells), *p* < 0.012, *post hoc* Tukey’s test). However, after repeated stress, the firing rate of place cells increased over the range of speed bins examined (PRE-Acute (*n* = 95 cells) vs. PRE-Chronic (*n* = 101 cells) *p* < 0.003, *post hoc* Tukey’s test) and this relationship further increased after experiencing the stress on day-10 (PRE-Chronic (*n* = 101 cells). vs. POST-Chronic (*n* = 88 cells), *p* < 0.0001, *post hoc* Tukey’s test). Circular histograms display the preferred phase of all place cells during theta **(C)**, FG **(D)**, and SG **(E)**. The thick line in each circular histogram depicts averaged phase across all cells.
