# Supplementary figures and images for: Differential Impact of Acute and Chronic Stress on CA1 Spatial Coding and Gamma Oscillations
Source: Front Behav Neurosci. 2021 Jul 20;15:710725. doi: 10.3389/fnbeh.2021.710725 (PMC8329706; doi:10.3389/fnbeh.2021.710725)

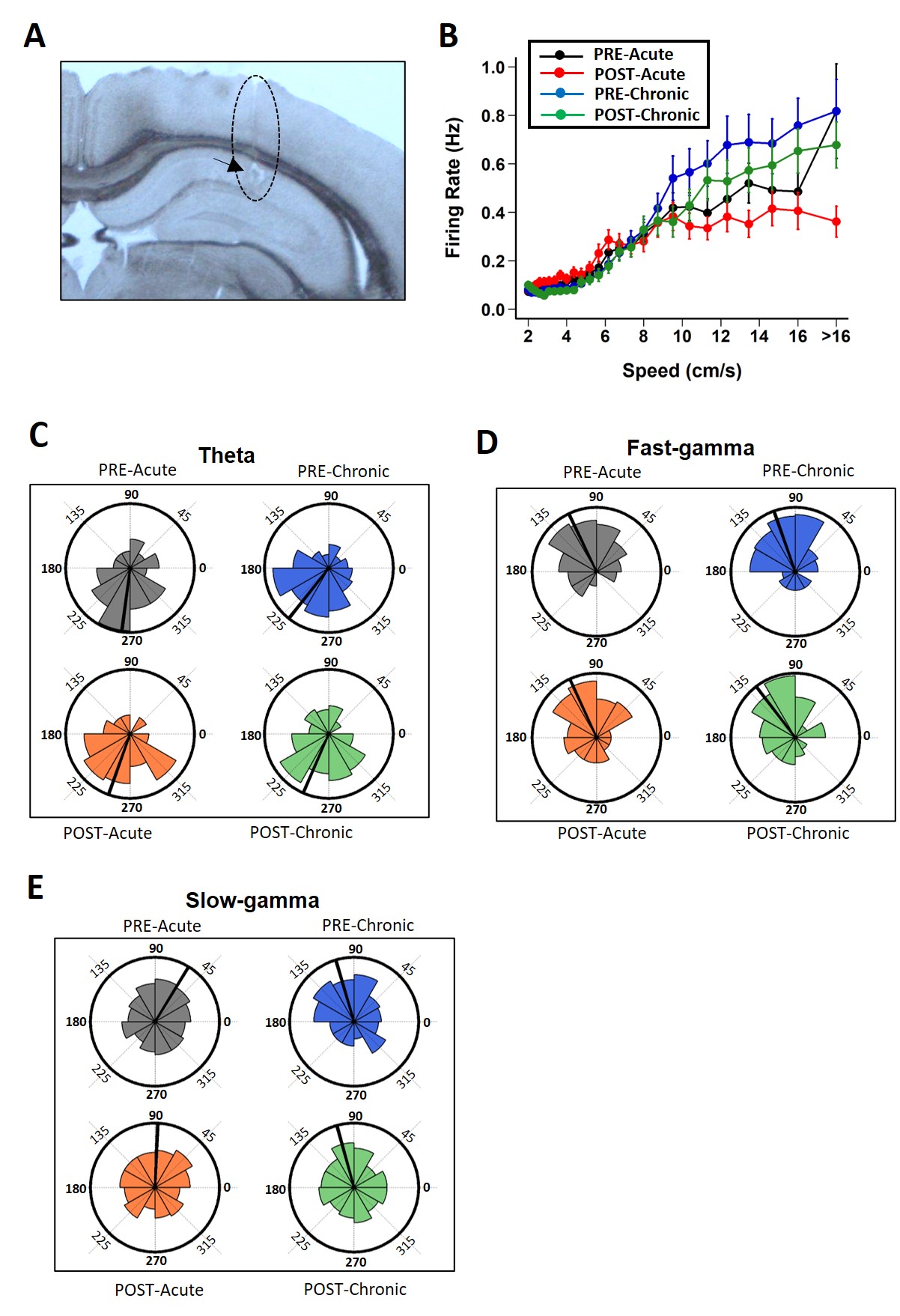

Supplement: SUPPLEMENTARY FIGURE 1 — The impact of stress on the relationship between CA1 place cell spiking vs. running speed and LFP oscillatory phase. (A) Coronal section of the hippocampus showing the tetrode locations (black arrows) at the CA1 pyramidal layer. (B) Dependence of firing rate on the speed was affected by stress (2-way mixed ANOVA: main effect of speed, F(1,370) = 1,424.752, p < 2.22 × 10^−16^, main effect of group, F(3,370) = 16.666, p = 3.546 × 10^−10^; interaction, F(3,370) = 37.866, p < 2.22 × 10^−16^, N = 5 mice). On day-1, after acute stress, place cells displayed lower firing over the range of speed bins examined (PRE-Acute (n = 95) cells vs. POST-Acute (n = 89 cells), p < 0.012, post hoc Tukey’s test). However, after repeated stress, the firing rate of place cells increased over the range of speed bins examined (PRE-Acute (n = 95 cells) vs. PRE-Chronic (n = 101 cells) p < 0.003, post hoc Tukey’s test) and this relationship further increased after experiencing the stress on day-10 (PRE-Chronic (n = 101 cells). vs. POST-Chronic (n = 88 cells), p < 0.0001, post hoc Tukey’s test). Circular histograms display the preferred phase of all place cells during theta (C), FG (D), and SG (E). The thick line in each circular histogram depicts averaged phase across all cells. [file Image_1.JPEG]
